# Comparative Community Ecology Reveals Conserved Ectoparasite Microbiomes Amidst Variable Host and Environment Microbiomes

**DOI:** 10.1002/ece3.71120

**Published:** 2025-04-02

**Authors:** Kelly A. Speer, Luis Víquez‐R, Winifred F. Frick, Ana Ibarra, Nancy B. Simmons, Katharina Dittmar, Ricardo Sánchez Calderón, Raisa Preciado, Rodrigo Medellín, Marco Tschapka, Simone Sommer, Susan L. Perkins

**Affiliations:** ^1^ Department of Biological Sciences Northern Arizona University Flagstaff Arizona USA; ^2^ Pathogen and Microbiome Institute Northern Arizona University Flagstaff Arizona USA; ^3^ Department of Biology Bucknell University Lewisburg Pennsylvania USA; ^4^ Institute of Evolutionary Ecology and Conservation Genetics Universität Ulm Ulm Germany; ^5^ Department of Ecology and Evolutionary Biology University of California Santa Cruz Santa Cruz California USA; ^6^ Bat Conservation International Austin Texas USA; ^7^ Laboratorio de Ecología y Conservación de Vertebrados Terrestres Universidad Nacional Autónoma de México Mexico City Mexico; ^8^ Department of Mammalogy American Museum of Natural History New York New York USA; ^9^ College of Arts and Sciences University at Buffalo Buffalo New York USA; ^10^ Escuela de Biología Universidad de Costa Rica San José California USA; ^11^ Facultad Interdisciplinaria de Ciencias Biológicas y de Salud Universidad de Sonora Hermosillo Sonora Mexico; ^12^ City College of New York City University of New York New York New York USA

## Abstract

The microbiome—the community of microorganisms that is associated with an individual animal—has been an important driver of insect biodiversity globally, enabling insects to specialize in narrow, nutrient‐deficient diets. The importance of maternally inherited, obligate bacterial endosymbionts in provisioning nutrients missing from these narrow dietary niches has been well studied in insects. However, we know comparatively little about the processes that dictate the composition of non‐maternally inherited bacteria in insect microbiomes, despite the importance of these bacteria in insect health, fitness, and vector competence. Here, we used two species of obligate insect ectoparasites of bats, the bat flies (Streblidae) *Trichobius sphaeronotus* and *Nycterophilia coxata*, to examine whether the microbiome, beyond obligate bacterial endosymbionts, is conserved or variable across geographic space, between ectoparasite species, or covaries with the external microbiome of their bat hosts or the cave environment. Our results indicate that ectoparasite microbiomes are highly conserved and specific to ectoparasite species, despite these species feeding on the blood of the same bat individuals in some cases. In contrast, we found high geographic variation in the fur microbiome of host bats and that the bat fur microbiome mimics the cave microbiomes. This research suggests that there is a constraint on blood‐feeding insect ectoparasites to maintain a specific microbiome distinct from their host and the environment, potentially to meet their nutritional needs. Given that many of these bacteria are not known to be maternally inherited, this research lays the foundation for future examinations of how blood‐feeding arthropods acquire and maintain bacteria in their microbiomes.

## Graphical abstract

Our results indicate that ectoparasite microbiomes are highly conserved and specific. In contrast, we found high geographic variation in host and environmental microbiomes, suggesting that there may be a constraint on blood‐feeding insect ectoparasites to maintain a specific microbiome.

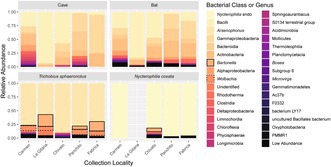

## 1 Introduction

Obligate bacterial endosymbionts have enabled insects to radiate and occupy previously inaccessible niches (Feldhaar and Gross 2009). Many insects rely on their bacterial microbiome for key aspects of development, nutrient acquisition, detoxification, immune function, and reproduction (Douglas 2009; Feldhaar and Gross 2009; Feldhaar 2011; Hansen and Moran 2014). For example, obligately symbiotic bacteria in true bugs (Hemiptera) enable their hosts to specialize in specific diets such as blood, phloem sap, xylem fluid, or seeds with toxic secondary metabolites (Sudakaran et al. 2017). In blood‐feeding insects, endosymbiotic bacteria are necessary to provision B vitamins that are missing from blood and that the insect cannot produce on its own (Douglas 2009). However, there are many members of the microbiome that are not obligately linked to their hosts, but that contribute to host immune function, health, and reproduction (Weiss and Aksoy 2011; Sassera et al. 2013; Wang et al. 2013).

Many facultative bacteria are undescribed taxa with unknown functions, and they may be variously acquired from the environment, host diet, or maternal inoculation (Yun et al. 2014). This broader microbiome community associated with blood‐feeding insects may act to affect a blood‐feeding insect's susceptibility to particular pathogens and downstream ability to vector those pathogens (Weiss et al. 2013; Weiss and Aksoy 2011). In pea aphids, facultative bacterial associates (e.g., those that are not obligately inherited) may provide greater protection against parasitic wasps or enhanced ability to access host plant resources (Simon et al. 2011). While studies on laboratory‐reared insects indicate that alteration of the microbiome community, including obligate symbionts and facultative bacteria, leads to decreased host immune function and survival (Cirimotich et al. 2011; Weiss et al. 2013), our understanding of the interactions between facultative bacteria and their hosts is much less developed than our understanding of those interactions between obligate endosymbiotic bacteria and their hosts. This disparity exists in part because we know so little about the systematics, function, acquisition (i.e., host colonization), and host‐specificity of facultative bacteria.

Here, we test the hypothesis that microbiomes, including obligate and facultative members, of blood‐feeding insects are strongly conserved instead of fluctuating with their environment. That facultative bacteria provide benefits to their host suggests that there may be selective pressure to maintain a microbiome with specific functional traits or taxonomic composition. If there is selective pressure acting to maintain the insect microbiome, we would expect microbiomes, either in their functional profile or taxonomic composition, to be highly conserved across the distribution of that insect species. If the microbiome is not constrained by its host insect, then we expect that the microbiome will mimic that of the environment where the insect lives, indicating no phylogenetic conservatism of microbiome taxonomic or functional profile. To test these hypotheses, we applied a comparative approach to examine the bacterial microbiome in two species of obligately blood‐feeding bat ectoparasites (Streblidae), the microbiomes of their host bat and the bat roost across a broad geographic area. The host‐associated life history strategy of bat flies narrows the suite of environmental sources from which these ectoparasites may acquire bacteria. Specifically, bat flies interact with the fur and blood of their bat host species and with the roost the bat occupies. Like all members of the Hippoboscoidea, bat flies are adenotrophically viviparous and leave the bat host to deposit a 3rd‐instar larvae on the roost substrate, where they pupate, emerge, and move across the roost to the appropriate host (Dick and Dittmar 2014). This provides opportunities for both the bat and roost to act as sources of variation in the bat fly microbiome if the bat fly microbiome is not constrained by the ectoparasite.

## 2 Materials and Methods

### 2.1 Sampling

**FIGURE 1:**
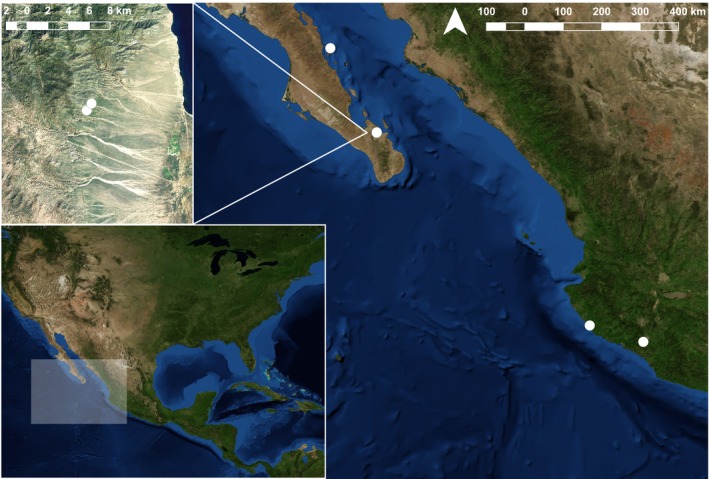
Map of sampling localities indicated with white dots. Upper left inset shows two proximal caves sampled from Baja California Sur (“Carmen” = Isla Carmen; Chivato & La Gitana are 1 km apart) and lower left inset shows the extent of the map.

Communities that are geographically distant may also be distinct from each other, especially when dispersal between sites is low (Cañedo‐Argüelles et al. 2015). We sampled bat flies associated with the migratory bat *Leptonycteris yerbabuenae* to minimize the impact of isolation‐by‐distance on bat fly microbiomes (Stoner et al. 2003; Cole and Wilson 2006). We sampled *L. yerbabuenae* from five localities in Mexico from November 2016 to November 2017, including three localities in Baja California Sur and two from Jalisco and Colima (Figure 1). Each year, *L. yerbabuenae* undertakes a seasonal migration to follow the blooming of agaves and cacti whose pollen and nectar are its primary food sources (Stoner et al. 2003). There are multiple migration routes followed by this species, but most mating behavior has been documented within a small subset of roosts in southern Mexico (Stoner et al. 2003; Laverty and Stoner 2022; Frick et al. 2018; Ceballos et al. 1997), where associated flies may also transfer between conspecific host individuals. Therefore, all of the sampled bats likely belong to a single population (Menchaca et al. 2020) and gene flow is expected to be high between the sampled caves.

All bats were collected using hand nets or mist nets directly outside or inside subterranean roosts and handled following guidelines provided by the American Society of Mammalogists for safe and humane handling of bats (Sikes et al. 2016). Bats were stored in individual clean cloth bags prior to sampling, and all bats were released within 6 h of capture. At collecting sites in Baja California Sur, the entire dorsum of each bat was swabbed using a sterile flocked‐cotton swab after bats were removed from cotton bags. At sites in Jalisco and Colima, bats were swabbed immediately following removal from the net, prior to being placed in the cotton bag. Swabs collected from Jalisco and Colima were lightly moistened with phosphate‐buffered saline prior to use, while swabs from sites in Baja California Sur were used dry. As differences in sampling protocol can impact which bacteria are detected, we used PERMANOVA to compare microbiomes from each swabbing method to examine possible bias resulting from differing methodologies (see details below). All swabs were placed directly in 95% ethanol for preservation. After removing bats from bags, bat flies ( *Trichobius sphaeronotus* and *Nycterophilia coxata*) were collected and placed directly into 95% ethanol. Cotton bags were cleaned prior to re‐use by washing in hot water, detergent, and OxyClean powder (Jalisco and Colima), followed by drying at high heat to minimize microbial contamination from the bags. Subterranean roost surfaces were swabbed in five different areas within the area of roosting *L. yerbabuenae* using five different sterile flocked‐cotton swabs (i.e., five swabs per roost). Roost swabs were preserved in 95% ethanol. Ethanol‐preserved ectoparasites and swabs were kept at room temperature for between 2 weeks and 1 month prior to being transferred to a −20°C freezer. All field work was conducted under the field collecting permit RAM: SGPA/DGVS/06361/17, with IACUC permits issued to W. Frick from University of California Santa Cruz (Frickw1602) and to K. Speer from the American Museum of Natural History (Speer 2016).

### 2.2 DNA Extraction, Library Preparation, and Sequencing

Prior to DNA extraction, bat flies were identified and sexed following (Wenzel 1976) and placed into individual sterile microcentrifuge tubes until ethanol evaporated (*~*5 min). To dilute external bacterial contamination, flies were washed twice by vortexing the specimen in 500 μL sterile phosphate‐buffered saline. Following washes, flies were punctured using sterile 0.25 mm pins (BioQuip, Rancho Dominguez, CA) to allow penetration of the exoskeleton by Proteinase K. Swabs were placed horizontally on the lip of sterile microcentrifuge tubes in a Biosafety Cabinet Class II to allow ethanol to evaporate while preventing cross‐contamination and laboratory contamination (*~*10 min). Negative controls were used to account for laboratory contamination during washing of flies and evaporation of ethanol from swabs. Swabs and flies were digested overnight using Proteinase K following the manufacturer protocol with Solid Tissue Buffer (Blue) (ZymoResearch, Irvine, CA). Digestion supernatant and swabs, but not cleared bat fly exoskeletons, were transferred to bead‐beating tubes for DNA extraction using the ZymoBIOMICS DNA Mini kit (ZymoResearch, Irvine, CA). Bead‐beating was performed either in a Disruptor Genie (Scientific Industries Inc., Bohemia, NY) for 20 min at 3000 rpm or in a Bead Mill Homogenizer (Fisher Scientific, Hampton, NH) for 4.5 min at 6 m/s in bouts of 45 s separated by 3 min breaks, following manufacturer instructions. The extraction protocol followed manufacturer instructions except: 500 μL of bead‐beat material was used instead of the recommended 400 μL, we increased the incubation time at the elution step from 1 min to 3 min, DNA was eluted using 100 μL sterile water (provided in kit) heated to 55°C, and DNA was eluted from the filter twice using the first eluate to rehydrate the filter for the second elution. DNA extractions were performed in a Biosafety Cabinet Class II to prevent contamination of samples; negative controls were used to account for contamination in kits, and a positive control (ZymoBIOMICS Microbial Community Standard) was used to examine bias in the DNA extraction protocol. To increase the concentration of DNA extractions, all samples and controls were evaporated in a bleached vacufuge in rounds of 48 samples. Samples were rehydrated with 30 μL sdH_2_O and stored at 4°C until library preparation. Library preparation of 16S rRNA amplicons followed recommendations from the Earth Microbiome Project (earthmicrobiome.org) and the Illumina 16S amplicon sequencing guide (see Supporting Information for details; Gilbert et al. 2010; Warinner et al. 2014; Apprill et al. 2015; Parada et al. 2016). Multiplexed samples were sequenced on the Illumina MiSeq using the Reagent Kit v3 (2 × 300bp) and 18% PhiX spike‐in by GENEWIZ (South Plainfield, NJ).

### 2.3 Contaminant Filtering, Data Transformation and Preprocessing

Samples were demultiplexed automatically by the Illumina BaseSpace software and loaded into the QIIME 2 v2019.1 pipeline for quality filtering (Bolyen et al. 2019). Raw sequences were trimmed (forward at 180 bp and reverse at 150 bp) and clustered into amplicon sequence variants (ASVs) using the DADA2 plugin (Callahan et al. 2016). Chimeras and PhiX spike‐in were simultaneously removed during ASV filtering. Taxonomy was assigned to ASVs using the classify‐sklearn naïve Bayes classifier (Bokulich et al. 2018) trained on the SILVA 132 99% identity 16S rRNA database (Quast et al. 2013), trimmed to hypervariable region four. Following taxonomic identification, all contaminant taxa from extraction, amplicon, and index negatives were removed from the sample data. In the amplicon negative, we detected 20 reads (out of a total 738) belonging to an *Arsenophonus*‐like endosymbiont prevalent in other samples. This endosymbiont is an expected member of the bat fly microbiome community, and its detection in the amplicon negative is likely due to minimal contamination prior to indexing or index bleed, a known issue when multiplexing samples (Kircher et al. 2012; Mitra et al. 2015; Eisenhofer et al. 2019); therefore, we did not remove *Arsenophonus* from the dataset. Next, we removed all Archaea, mitochondria, chloroplasts, and known laboratory contaminants (Eisenhofer et al. 2019). To identify additional contaminants not detected in the negative controls, we used the package *decontam* implemented in RStudio v3.6.0 to detect ASVs that increased in frequency as initial library concentration decreased, an indicator that an ASV is a contaminant (Team and Others 2015; Davis et al. 2018). Eleven ASVs were flagged by *decontam* and removed. Positive controls of extraction and amplification protocols contained all expected members of the community, with the exception of the amplification positive, which was missing reads from *Pseudomonas aeruginosa* . All taxa present in the positive controls were removed from the rest of the dataset. Lastly, we removed 11 bat fly samples whose microbiomes were not at least 40% comprised of *Arsenophonus*, *Arsenophonus*‐like endosymbionts, *Bartonella*, and *Wolbachia*. These bacteria are expected to dominate the composition of bat fly microbiomes (Wilkinson et al. 2016; Speer et al. 2022), and samples that are missing large proportions of these taxa are likely highly contaminated. Following the removal of contaminant ASVs or highly contaminated samples, we used the R package *phyloseq* to further quality filter and transform the data (McMurdie and Holmes 2012). We removed samples that fell below a minimum sequencing depth of 1000 reads, and any ASV that had fewer than 5 reads detected in a single sample was removed from that sample.

To account for the compositionality of high‐throughput metabarcoding data, where the abundance of bacteria detected in each sample is not reflective of the true number of bacteria in the original sample and is not independent of other samples sequenced in the same flow cell (Pawlowsky‐Glahn et al. 2015; Tsilimigras and Fodor 2016; Gloor et al. 2017; Silverman et al. 2017), we use log‐ratio transformations. Log‐ratio transformations standardize the abundance of a microbe within a sample by the abundance of other microbe(s) in the dataset, allowing comparisons using multivariate statistics (Gloor et al. 2017; Silverman et al. 2017). We use the phylogenetic isometric log‐ratio transform in the *philr* package for R, which uses the phylogenetic relationships between the bacteria present in a sample to transform abundances into an unconstrained coordinate system (Silverman et al. 2017). To reconstruct the phylogeny, we use the mafft plugin in QIIME 2 to align all ASVs (Katoh and Standley 2013) and masked highly variable regions. The fasttree2 plugin was used to estimate the phylogeny with default parameters (Price et al. 2010). The *philr* package first resolves polytomies, which are incompatible with *philr*, using the R package *ape* to insert tips of branch length zero (Paradis and Schliep 2018). Then *philr* introduces a pseudo count of 1 to all samples, weights taxa by their Euclidean norm times geometric mean across samples to minimize the impact of low abundance taxa, and uses branch‐length weighting to scale transformations by their phylogenetic distance (option “square root of the sum of children's branch lengths” Silverman et al. 2017).

### 2.4 Bar Plots and Ordination

We constructed bar plots of average relative abundance of bacterial taxa within each sample type and within sample type split by collection locality using the *viridis* color palette and *ggplot2* (Wickham 2016; Garnier 2018). Bacteria were clustered by genus for bat flies and by phylum for cave swabs and bat swabs. Genera or phyla occurring at < 1% relative abundance in each sample type within each locality were combined into a “Low Abundance” group. Samples that could not be identified to genus or phylum were clustered into an “Unidentified” group.

Differences in microbial communities by sample type and collection locality were visualized with Principal Coordinates Analysis, implemented in *phyloseq*, on Euclidean distance between *philr*‐transformed abundances. We used a Permutational Multivariate Analysis of Variance (PERMANOVA) to test whether sample type (i.e., bat fly species, bat swab, or cave swab), bat fly sex, bat host sex, bat individual from which a bat fly was collected, and collection locality are correlated with microbial community composition, with 9999 permutations (*adonis* command in the R package *vegan*; Anderson 2014). PERMANOVA is a statistical tool used to assess differences in multivariate datasets across multiple groups. PERMANOVA evaluates groups of objects, testing whether the centroids and dispersion across groups are equivalent under the null hypothesis. When sampling is uneven between groups, PERMANOVA is sensitive to heteroscedasticity. We tested for homogeneity of dispersion of each explanatory variable using *betadisper* permuted 999 times with *permutest* (R package *vegan*; Oksanen et al. 2018).

### 2.5 Random Forest Classification

As many of our categorical variables were heteroscedastic and contained uneven sample sizes, we used random forest classification to examine the robustness of our PERMANOVA findings. Random forests are an ensemble of machine‐learning algorithms that rely on repeated construction of decision trees based on a random subsampling with replacement of the training data. Random forests perform well when data are categorical, and the algorithm does not make a priori assumptions about the underlying distribution of the data (Breiman 2001; Cutler et al. 2007). We examined how accurately sample type (e.g., cave swab, bat swab, *Trichobius sphaeronotus* , or *N. coxata*), collection locality (total dataset and bat swab only dataset), and bat fly sex (within the *T. sphaeronotus* dataset and within the *N. coxata* dataset) can be distinguished by microbiome community composition.

We used *~*80% of the data to train a random forest model in the R package *caret* (Kuhn et al. 2018), taking care to include 80% of each class within the outcome variable. We used the *ranger* method to fit the model, which relies upon the package *e1071* (Wright and Ziegler 2015; Meyer et al. 2019). We estimated the accuracy of training models with different tuning parameters, incorporating five replicates of 10‐fold repeated cross‐validation. We selected the model with the highest Kappa (accuracy accounting for sample size imbalance between classes) for testing against the remaining 20% of data that was unseen by the model during training. The accuracy of the predicted classifications of the outcome variable by the model on the test data was assessed using a confusion matrix, from which precision, recall, and Kappa were calculated. When training a model to predict sample type, cave swabs were up‐sampled by resampling this class of samples multiple times with replacement to increase the representation of cave swabs in the training dataset. Similarly, males within the *N. coxata* dataset were up‐sampled when training a model to predict the sex of the flies.

Predictor variables changed based on the outcome variable, but the microbiome community was always represented by two predictor variables—the vectors from Axis 1 and 2 of the PCoA corresponding to the dataset being examined. The complete dataset was used for predicting sample type and collection locality. Collection locality was included as a predictor variable when sample type was the outcome, while sample type was a predictor when collection locality was the outcome. We also used collection locality as the outcome variable on a bat swab‐only dataset, where bat sex was a predictor variable in addition to microbiome composition. Fly sex was used as the outcome variable in two models, one trained on a *T. sphaeronotus* ‐only dataset and one trained on a *N. coxata*‐only dataset. For these models, collection locality was used as a predictor variable on top of microbiome composition.

## 3 Results

Of 192 prepared libraries, 163 libraries were used for downstream analysis after quality filtering. Filtered libraries ranged in sequencing depth from 1009 to 16,431 quality‐filtered reads. Quality filtering resulted in a dataset of 163 samples (cave swabs = 5, bat swabs = 42, bat flies = 116) with 6027 detected bacterial taxa (cave swabs = 609 ASVs, bat swabs = 4050 ASVs, bat flies = 1746 ASVs). Bat flies were dominated by Gammaproteobacteria in the Enterobacteriaceae, while cave and bat swabs were more diverse. The occurrence of bat fly species on swabbed bats is summarized in Table S1.

### 3.1 Differentiation Between Microbiomes

**FIGURE 2:**
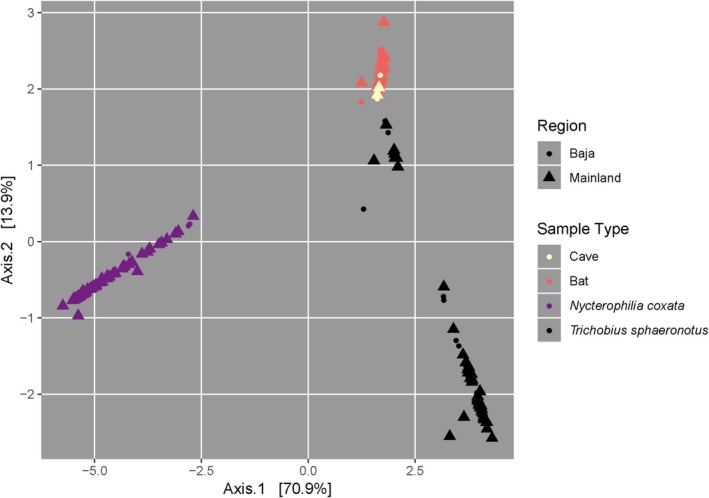
PCoA of microbiome samples colored by sample type, with circles indicating samples collected from Baja California Sur and triangles indicating samples collected from mainland Mexico.

Principal coordinates analysis and PERMANOVA indicate that cave swabs and bat swabs have distinct microbiomes from both *N. coxata* and *T. sphaeronotus* , and that the microbiomes of the two fly species are distinct from each other (Table 1, Figure 2). Collection locality and broader geographic region (i.e., mainland vs. Baja California Sur) play a significant role in differentiating bat and cave microbiome communities. Both sample type and collection locality violate the PERMANOVA assumption of homoscedasticity, so significance may be driven by variation in sample sizes.

**TABLE 1:** PERMANOVA results indicating the *p*‐value, *R* ^2^, and *p*‐value for homoscedasticity (significance indicates a violation of the assumptions of PERMANOVA). Results are provided for the full dataset, the dataset containing only bat swabs and cave swabs, a dataset containing only *Nycterophilia coxata* samples, a dataset containing only *Trichobius sphaeronotus* samples, and a dataset where endosymbionts have been removed in silico from bat fly microbiomes. Gray shading indicates explanatory variables that significantly differentiated microbiomes and did not violate the assumption of homoscedasticity. Significance is indicated by asterisks (*0.05–0.01, **0.009–0.001, ***0.0009–0.0001) and a period indicated near significant (0.055–0.094).

	Explanatory variable	PERMANOVA *p*	*R* ^2^	Homoscedasticity *p*
Complete dataset	Sample Type (Cave, Bat, *N. coxata*, *T. sphaeronotus*)	0.0001***	0.796	0.041*
	Collection locality	0.003**	0.074	0.001**
	Baja/Mainland	0.005**	0.038	0.001**
Bat swabs + cave swabs	Sample type (Cave, Bat)	0.1839	0.028	0.131
	Collection locality	0.0001***	0.217	0.079
	Baja/Mainland	0.0001***	0.092	0.579
	Bat sex (Bat‐only dataset)	0.3623	0.026	0.56
*Nycterophilia coxata*	Collection locality	0.0604	0.101	0.367
	Baja/Mainland	0.0618	0.042	0.068
	Bat fly sex	0.1121	0.035	0.36
	Bat sex	0.4781	0.012	0.76
	Bat individual	0.1618	0.028	0.151
	*Bartonella*, *Wolbachia*, or Undetected	0.0283*	0.091	0.143
	Bat fly sex + Bartonella/Wolbachia infection	0.0990, 0.0195*	0.035, 0.101	see above
*Trichobius sphaeronotus*	Collection locality	0.6019	0.057	0.794
	Baja/Mainland	0.4138	0.015	0.803
	Bat fly sex	0.0011**	0.159	0.003**
	Bat sex	0.5917	0.009	0.262
	Bat individual	0.1586	0.032	0.076
	*Bartonella*, *Wolbachia*, Co‐infection, or Undetected	0.0002***	0.281	0.001**
	Bat fly sex + Bartonella/Wolbachia infection	0.0001***, 0.0006***	0.159, 0.214	see above
No endo.	Sample type (Cave, Bat, *N. coxata*, *T. sphaeronotus*)	0.0001***	0.237	0.001**
	Collection locality	0.0001***	0.077	0.272
	Baja/Mainland	0.0001***	0.027	0.288

**FIGURE 3:**
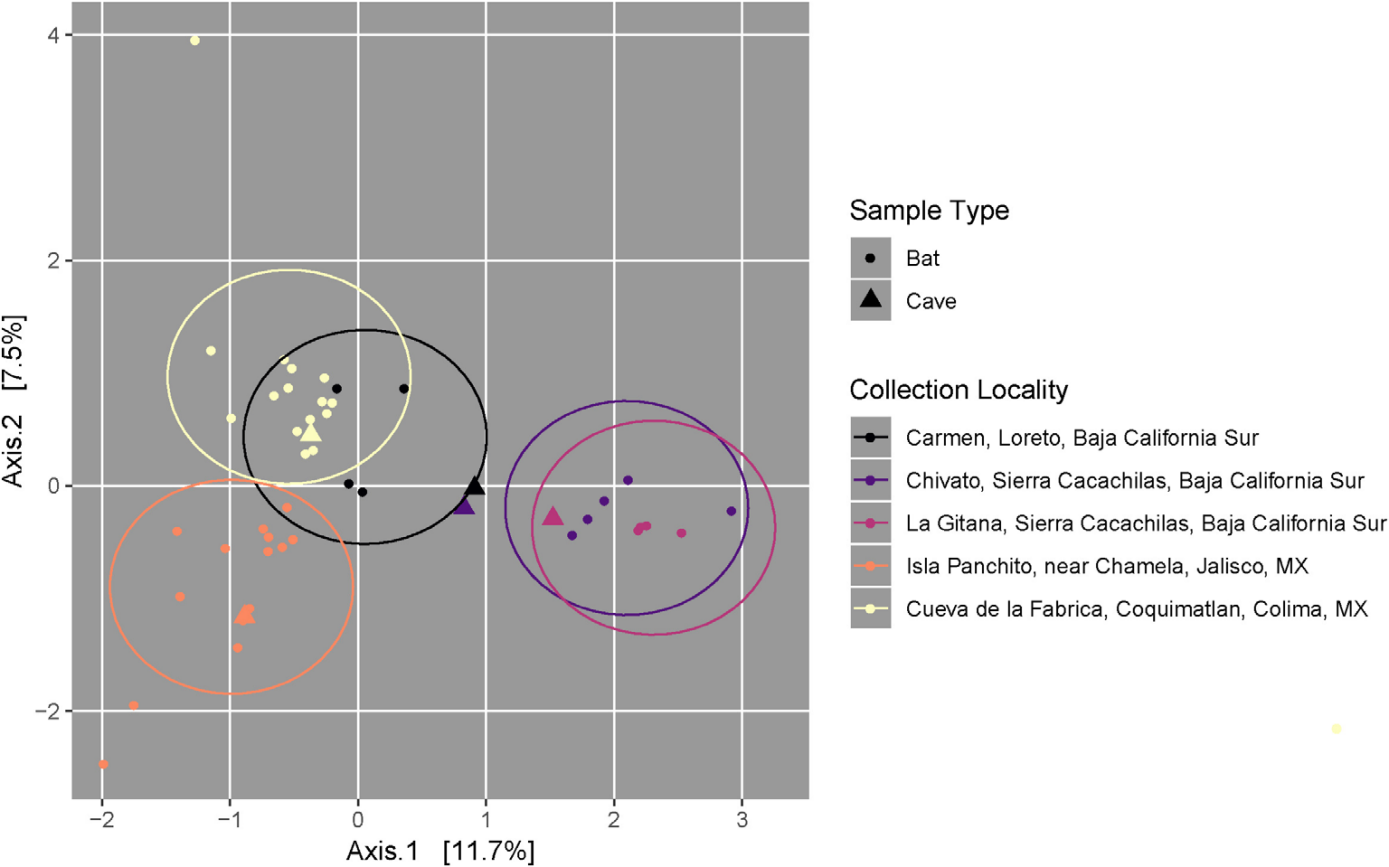
PCoA of bat (circles) and cave (triangles) samples only, colored by collecting locality. Ellipses indicate all samples within an even Euclidean distance from the center of the group. Euclidean distance was set to 0.95.

When examining bat fur and cave microbiomes, collection locality significantly contributes to microbiome differentiation (Table 1, Figure 3). La Gitana and Chivato, which are the geographically closest sites, have overlapping bat fur and cave microbiomes when plotted in principal coordinate space. Isla Panchito and Cueva de la Fábrica, which are the two mainland localities, have microbiomes that are different from each other and from the southernmost Baja localities (Chivato and La Gitana), but bats and cave swabs from Isla Panchito (Jalisco) and Isla Carmen (Baja Sur California) have similar microbiomes. When collection locality and geographic region are analyzed sequentially to account for their nestedness, collection locality explains all of the variation. There is no significant differentiation between the bat sexes in their fur microbiomes.

Within *N. coxata*, collection locality, region, and infection with *Bartonella* or *Wolbachia* are significant explanatory variables for differentiation between sampled microbiomes (Table 1, Figure S2). Similar to the complete dataset and the bat swab and roost data, when collection locality and region are examined sequentially, collection locality accounts for all of the variation attributable to these two variables. There is not a statistically meaningful impact of bat fly sex, bat sex, or individual bat on the microbiomes of *Nycterophilia*.

In contrast, bat fly sex is an important variable driving the microbiome composition of *T. sphaeronotus* , while collection locality and region had no significant contribution (Table 1, Figure S3). Similar to *Nytererophilia*, we found that *Bartonella* and *Wolbachia* infections were important factors in differentiating individuals within *Trichobius*. Many samples within *T. sphaeronotus* were infected with both *Wolbachia* and *Bartonella*, but there were no co‐infections detected in *Nycterophilia*. Both bat fly sex and *Bartonella* and *Wolbachia* infection status violated PERMANOVA assumptions of homoscedasticity. Bat sex and bat individuals had no significant impact on *Trichobius* microbiome clustering.

When we remove *Arsenophonus* and the *Nycterohilia*‐specific endosymbiont, PERMANOVA indicates that sample type and collection locality remain important explanatory variables for microbiome variation (Figure S4). However, ellipses showing 95% confidence intervals and distance from the center of the group indicate no differentiation between cave swabs, bat swabs, and *Nycterophilia*. *Trichobius* is readily differentiated from the other sample types.

### 3.2 Classifying Samples by Their Microbiome Ordination

To examine the explanatory power of variables that violated the assumptions of PERMANOVA, we used random forests to classify samples by these explanatory variables using the principal coordinate axis vectors for each sample. Random forests indicated that sample type in both the complete dataset and in the dataset lacking insect primary endosymbionts can be classified using the microbiome principal coordinate axis loadings (Table 2). Collection locality in the bat‐only dataset was accurately classified by the model. While classifying the sample type of the test dataset lacking insect endosymbionts, cave swabs were mistaken for bat swabs and some *Trichobius* samples were mistaken as *Nycterophilia*, but no swabs were mistaken for insects and no insects were mistaken for swabs. When endosymbionts were present in the data, *Trichobius* and *Nycterophilia* were not misclassified by the model. In the bat‐only test dataset, collection locality was never misclassified. Models trained to classify other variables were not more accurate than a model that always chooses the category with the largest sample size (No Information Rate), indicating those variables are not strongly predicted by microbiome community composition.

**TABLE 2:** Random Forest classification of categorical variables based on microbiome principal coordinate axis 1 and axis 2. Accuracy of the model in predicting the classes of each variable in the test data set, the “No Information Rate” where the accuracy of the model is calculated for choosing only the class with the largest sample size, *p*‐value of accuracy being greater than the “No Information Rate,” and Kappa are provided for assessing the strength of the signal in the data. Gray shaded rows indicated variables where the model performs significantly better than the “No Information Rate.”

Dataset	Explanatory variable	Accuracy	NIR	*p*	Kappa
Complete	Sample type	0.968	0.355	< 0.0001	0.952
	Collection locality	0.300	0.400	0.9060	0.016
Bat swabs	Collection locality	1.000	0.500	0.0156	1.000
	Bat sex	0.750	0.875	0.9327	−0.143
*Nycterophilia coxata*	Collection locality	0.636	0.455	0.1819	0.436
	Bat fly sex	0.727	0.636	0.3883	0.441
	*Bartonella* and *Wolbachia* infection	0.909	0.818	0.3788	0.621
*Trichobius sphaeronotus*	Collection locality	0.750	0.875	0.9327	−0.143
	Bat fly sex	0.455	0.546	0.8181	−0.100
	*Bartonella* and *Wolbachia* infection	0.667	0.667	0.6503	0.182
No endosymbionts	Sample type	0.871	0.355	< 0.0001	0.808
	Collection locality	0.433	0.422	0.4215	0.164

### 3.3 Bacterial Relative Abundance by Sample Type and Collection Locality

The microbiomes of roosts, bat fur swabs, and their ectoparasitic flies are each dominated by different bacteria (Figure S1). Both bat fur and roosts had large portions of Pseudomonadota (formerly Proteobacteria), Basillota (formerly Firmicutes), Actinomycetota (formerly Actinobacteria), and Bacteroidota (formerly Bacteroidetes). Bat fly microbiomes are dominated by their primary endosymbionts. In *T. sphaeronotus* , the primary endosymbiont is *Arsenophonus*, which also acts as the primary symbiont across many other bat fly species within the Streblidae and Old World Nycteribiidae (Trowbridge et al. 2006; Morse et al. 2013; Speer et al. 2022). In *N. coxata*, the primary endosymbiont is a previously sequenced bacterium in the Enterobacteriaceae that has not been described but is unique to the genus *Nycterophilia* (Morse et al. 2012). While there is little variation between female and male *N. coxata*, there are qualitative differences between female and male *T. sphaeronotus* . Male *T. sphaeronotus* appears to have a higher proportion of *Bartonella* and *Wolbachia* than female *T. sphaeronotus* . There is a very low abundance of both *Bartonella* and *Wolbachia* in *N. coxata*.

**FIGURE 4:**
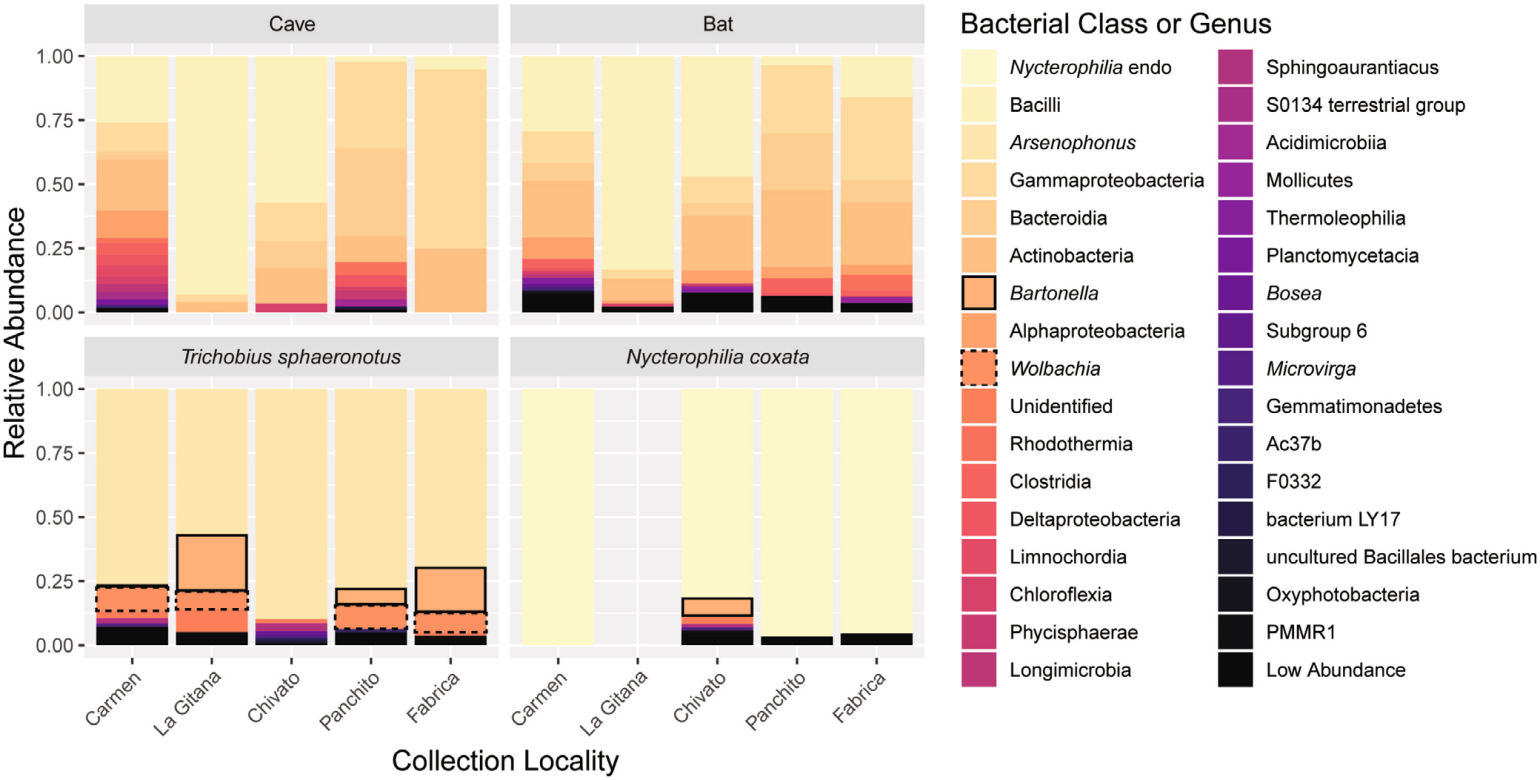
Average relative abundance with each bar representing a sampling locality, clustered by the sample type. In cave swabs and bat swabs, colors correspond to class of bacteria, while in bat flies, colors correspond to genus of bacteria. *Bartonella* relative abundance is indicated with a bold black outline and *Wolbachia* relative abundance is indicated with a dashed black outline.

When split by collection locality, cave and bat swabs remain similar to each other within a site and between sites (Figure 4). Specifically, at Panchito, where the abundance of Firmicutes in the cave is lowest, the abundance of Firmicutes detected from the bat swabs is also lowest. The La Gitana site showed a greater relative abundance of Firmicutes in both the cave and bat swabs compared to other sites. The average microbiome composition detected from cave swabs and bat swabs in Baja Californa Sur and on the mainland did not differ despite the use of variable swabbing protocols between these groups of sites. In bat flies, the relative abundance of *Wolbachia* and *Bartonella* is extremely low in *T. sphaeronotus* at the Chivato site, but is higher than at other sites in *N. coxata*. This is likely an artifact of the samples we collected at this site—only 1 *Trichobius* female was collected and only *Nycterophilia* males were collected there.

## 4 Discussion

### 4.1 The Microbiome of Bat Flies Is Distinct From Their Environment

Here, we tested whether the microbiome of bat flies was conserved across geographically distant localities and within bat fly species occupying the same bats and roosts. We found that bat fly microbiomes were distinct from their environment. It is possible that a greater proportion of insect microbiomes than previously understood is the result of maternal or population inoculation, creating the possibility of greater coevolution between insects and their microbiomes beyond primary and secondary symbionts. Our results provide support for the hypothesis that blood‐feeding insects maintain a specific microbiome community and may selectively filter facultatively associated bacteria. Bat flies have conserved, host‐specific microbiomes across sampling localities, in contrast to the microbiomes of bat fur and roosts, both of which vary geographically (Tables 1 and 2, Figures 3 and 4). The strongest factor differentiating bat fly microbiomes from their environment (i.e., cave swabs and bat swabs) is the large relative abundance of primary endosymbionts; however, bat fly microbiomes remain distinct from environmental microbiomes even when primary endosymbionts are removed from the data (Tables 1 and 2). This suggests that bat flies either inherit more of their microbiome than previously known, filter the bacteria that colonize their internal microbiomes, or some combination of these processes.

### 4.2 Environmental Contribution to the Microbiome of Bat Flies

The observation that the microbiomes of *N. coxata* and *Trichobius sphaeronotus* become difficult to differentiate following the removal of their primary symbionts from the data (Table 2) suggests that direct transmission cannot completely account for the bat fly microbiome. The environment is likely contributing bacteria to the microbiome of bat flies, but the bacteria that successfully colonize the bat fly microbiome may be filtered through fly immune responses, priority effects with bacteria that previously colonized the fly, or fly microclimatic preference. For example, within caves, topology may create areas of specific microclimates that harbor unique bacterial communities. These microclimatic niches are hypothesized to contribute to the separation of bat species within the same cave (Brunet and Medellín 2001) and also in the ability of host‐specific bat flies to locate an appropriate bat host after emerging from a puparium on the cave wall (Patterson et al. 2007; Dittmar et al. 2009). Future work should swab pupal deposition sites to examine variation in the cave microbiome and attempt to quantify microclimatic variation.

While primary and secondary endosymbionts are maternally inherited (vertical inheritance), bacteria may also be acquired from other host individuals in the same population or selectively acquired from their environment (horizontal transmission; Scheuring and Yu 2012). Bat fly microbiomes do not vary by collection locality in contrast to the fur microbiome of their bat hosts and the roost microbiome, suggesting that a greater proportion of the fly microbiome may be either vertically or horizontally transmitted from other members of the insect population than previously understood. Given the limitations of 16S rRNA to identify bacteria, we do not have the ability to test the genetic similarity of the bacteria within each sample type (Mignard and Flandrois 2006). Future research on the genetic diversity of bacteria found within a single population of individual flies that use a greater genomic sampling of loci would help illuminate which other bacteria may be transmitted between bat flies in this system.

### 4.3 Bat Fly Microbiomes May Differentially Interact With *Wolbachia* and *Bartonella*

Microbiome communities influence the susceptibility of a host insect to disease (Weiss and Aksoy 2011), which has downstream implications for the competence of that insect to transmit the disease and act as a vector. Bat flies are known vectors of *Polychromophilus*, a bat‐specific malaria‐like pathogen, and may vector *Bartonella* to their host bats, where it may act facultatively or pathogenically (Obame‐Nkoghe et al. 2016; Stuckey et al. 2017). *Wolbachia* may act facultatively or as a reproductive parasite of insects, causing male sterility or death to advance its own prevalence in a population (Cariou et al. 2017). The observation that most *Trichobius* in our study were infected with both *Bartonella* and *Wolbachia*, but most *Nycterophilia* were not infected with either, suggests that these two fly species may be differentially susceptible despite occupying the same bat host and the same roosts (Table 1; Figures S2 and S3). Future work is needed to link pathogen prevalence in bats with bat fly microbiomes, but bat fly microbiomes may be an important factor in determining arthropod‐vectored pathogen prevalence in bats.

### 4.4 Geographic Variation in Bat Fur and Cave Microbiomes Despite High Dispersal

As *Leptonycternis yerbabuenae* is a migratory bat species, we had expected to find homogenous fur microbiomes for this taxon across our four sampling localities, which are positioned along their route of annual seasonal migration. Our original predictions were incorrect in this respect—the fur microbiomes of *L. yerbabuenae* were specific to collection site, with geographically proximal sites having more similar microbiomes (Tables 1 and 2, Figure 3). Similarly, a previous study by our team showed that *L. yerbabuenae* had strong locality and biome signatures in their microbiome, but with large differences between biomes and moderate similarity between two of our sampling sites (Chamela and Coquimatlán) (Víquez‐R et al. 2021). Previous work on human microbiomes suggests that microbial communities can change rapidly, with gut microbiomes changing over the course of days in response to diet (David et al. 2014). Our results suggest that bat fur microbiomes may also change rapidly in response to bat roosting and foraging sites. Future research should examine the ways the environmental specificity of the bat fur microbiome may change in response to bactericide or fungicide use in agricultural land, roost alterations (such as guano removal), differences in roost sites (e.g., natural caves vs. man‐made structures), and land use change.

While it is well documented that insects with narrow diets are reliant on maternally inherited primary endosymbiotic bacteria, the sources of other members of the microbiome in these insects are not well understood. From previous work in mosquitoes and ticks, we know that the microbiome of blood‐feeding arthropods can change through ontogeny, presumably due to varying environmental interactions (Duguma et al. 2015; Zolnik et al. 2016). This suggests that the environment is an important source of bacteria for arthropod microbiomes (Yun et al. 2014). However, it is unclear whether selection acts to narrow the pool of bacteria present in insect microbiomes (Foster et al. 2017), leading to reduced geographic variation in the microbiome within an insect species. The results presented here demonstrate the need for further work to explore whether and how selection may be acting to constrain non‐maternally inherited bacteria of blood‐feeding insects.

## Author Contributions

**Kelly A. Speer:** conceptualization (lead), data curation (equal), formal analysis (lead), funding acquisition (equal), investigation (lead), methodology (lead), project administration (lead), resources (supporting), software (lead), visualization (lead), writing – original draft (lead), writing – review and editing (lead). **Luis Víquez‐R:** conceptualization (equal), funding acquisition (equal), investigation (equal), methodology (equal), project administration (equal), resources (equal), writing – review and editing (equal). **Winifred F. Frick:** data curation (equal), investigation (equal), methodology (equal), project administration (equal), writing – review and editing (equal). **Ana Ibarra:** investigation (equal), methodology (equal), writing – review and editing (equal). **Nancy B. Simmons:** conceptualization (supporting), methodology (supporting), resources (supporting), supervision (equal), writing – review and editing (equal). **Katharina Dittmar:** conceptualization (equal), investigation (supporting), methodology (equal), writing – review and editing (supporting). **Ricardo Sánchez Calderón:** investigation (equal), methodology (supporting), writing – review and editing (supporting). **Raisa Preciado:** investigation (supporting), methodology (supporting), writing – review and editing (supporting). **Rodrigo Medellín:** conceptualization (supporting), methodology (supporting), writing – review and editing (supporting). **Marco Tschapka:** conceptualization (supporting), resources (supporting), supervision (equal), writing – review and editing (supporting). **Simone Sommer:** conceptualization (supporting), methodology (supporting), resources (equal), supervision (equal), writing – review and editing (equal). **Susan L. Perkins:** conceptualization (equal), funding acquisition (equal), methodology (equal), resources (equal), supervision (equal), writing – review and editing (supporting).

## Conflicts of Interest

The authors declare no conflicts of interest.

## Supporting information


Data S1

## Data Availability

Raw sequencing data are available through NCBI SRA (BioProject PRJNA1223959) and the code used for data analysis is available at https://github.com/SPEER‐lab/ComparativeMicrobiomics_Bats_BatFlies_Caves_Mexico (Doi: https://zenodo.org/records/14873485).
