# Supplementary material for: Comparative Community Ecology Reveals Conserved Ectoparasite Microbiomes Amidst Variable Host and Environment Microbiomes
Source: Ecol Evol. 2025 Apr 2;15(4):e71120. doi: 10.1002/ece3.71120 (PMC11962207; doi:10.1002/ece3.71120)

**Supplementary Information for “Comparative community ecology reveals conserved ectoparasite microbiomes amidst variable host and environment microbiomes.”**

Kelly A. Speer^*^, Luis Víquez-R, Winifred F. Frick, Ana Ibarra, Nancy B. Simmons, Katharina Dittmar, Ricardo Sánchez Calderón, Raisa Preciado, Rodrigo Medellín, Marco Tschapka, Simone Sommer, and Susan L. Perkins

*Corresponding author: Kelly A. Speer, kelly.speer@nau.edu

**DNA Library Preparation**

Each sample was amplified in triplicate for 16S hypervariable region 4 using the primers 515f and 806r with Illumina overhang adapter sequences (Gilbert et al. 2010; Warinner et al. 2014; Apprill et al. 2015; Parada et al. 2016). Amplicon PCRs were conducted in 25μL volumes containing 10μL 5PRIME HotMasterMix (final concentrations of 1U Taq DNA Polymerase, 45mM Cl, 2.5mM Mg_2_+, 200μM of each dNTP; Quantabio, Beverly, MA), 5μL of each primer at 1μM concentration (final concentration of 0.2μM each), and 5μL of template DNA. Thermocycler conditions used an initial denaturation of 94°C for 2min, followed by 30 cycles of 94°C for 20s, 55°C for 30s, and 65°C for 30s, with a final elongation at 65°C for 5min. Prior to amplification, cave swabs were pooled by collection locality and all extraction negative controls were pooled together. An additional negative control and positive control (ZymoBIOMICS Microbial DNA Standard) were included in the amplification step. Triplicate amplicons were pooled and cleaned using a 1:1 ratio of PCR to AMPure XP beads following manufacturer protocol (Beckman Coulter, Indianapolis, IN). A representative subsample of cleaned amplicons were examined using the DNA HS Assay kit for Qubit 2.0 fluorometry (Invitrogen, Waltham, MA) and Bioanalyzer 2100 DNA High Sensitivity Reagents (Agilent, Santa Clara, CA) to check quality. To barcode amplicons, a secondary PCR was conducted in 50 μL volumes containing 25μL KAPA HiFi HotStart Ready Mix (final concentrations of 0.5U Taq DNA polymerase, 2.5mM MgCl_2_, and 0.3mM of each dNTP; Roche, Basel, Switzerland), 5μL of each the forward and reverse indexing primers (Illumina Nextera XT Index Kit v2, set A and set C), and 5μL clean amplicon PCR product. Thermocycler conditions included an initial denaturation at 95°C for 3min, 8 cycles of 95°C for 30s, 55°C for 30s, and 72°C for 30s, with a final elongation at 72°C for 5min. Index PCRs were cleaned and quality checked as with amplicon PCRs, except the concentrations of all samples were estimated using Qubit 2.0 fluormetry. Samples were diluted to 2.5nM and 3μL of each indexed sample was pooled. There were 6 samples, ranging in concentration from 0.4-2.21nM, that were not diluted and were added to the pool in greater volumes corresponding to their concentrations. Twelve samples failed library preparation with a concentration less than 0.21nM. These were used to dilute the pooled successful samples to 2nM by adding 20uL of each failed sample, in the hopes of gaining some data from these samples.

Supplementary Table 1: Sample type information. The ID number, date collected, collection locality, sex of *Leptonycteris yerbabuenae* from which swabs and bat flies were collected, and the sex and number of each bat fly that were collected are summarized.

| **ID No** | **Date Collected** | **Collection Locality** | **Bat Sex** | ***Trichobius sphaeronotus*** | ***Nycterophilia coxata*** | **Bat Swab** |
| --- | --- | --- | --- | --- | --- | --- |
| 249723 | 27-May-17 | Carmen, Loreto, Baja California Sur | female |  | 1M |  |
| 249733 | 27-May-17 |  | female | 1F | 1F | Y |
| 249743 | 27-May-17 |  | female | 2F 1M |  | Y |
| 249753 | 27-May-17 |  | female | 2M | 1F | Y |
| 249773 | 27-May-17 |  | female | 1F 1M |  | Y |
| 249783 | 27-May-17 |  | female | 1F 1M |  | Y |
| 249705 | 29-May-17 | La Gitana, Sierra Cacachilas, Baja California Sur | female | 1F | 1F | Y |
| 249715 | 29-May-17 |  | female | 1F 1M |  | Y |
| 249725 | 29-May-17 |  | female | 1F 2M |  | Y |
| 249735 | 29-May-17 |  | female | 1F |  | Y |
| 249744 | 29-May-17 |  | female | 1F 1M |  | Y |
| 249755 | 29-May-17 |  | female | 2F |  |  |
| 249785 | 29-May-17 |  | female | 1F 1M |  |  |
| 249696 | 30-May | Chivato, Sierra Cacachilas, Baja California Sur | male | 1M |  | Y |
| 249706 | 30-May |  | male | 1F |  | Y |
| 249716 | 30-May |  | female | 1M |  | Y |
| 249726 | 30-May |  | female | 1F 1M |  | Y |
| 249746 | 30-May |  | female |  | 3M | Y |
| 249756 | 30-May |  | female |  | 3M |  |
| 249766 | 30-May |  | male |  | 2M |  |
| 249683 | 13-Dec-16 | Cueva de la Fábrica, Coquimatlán, Colima, MX | male | 1F 1M | 1F 1M |  |
| 249684 | 13-Dec-16 |  | male |  | 4F |  |
| 249685 | 13-Dec-16 |  | male | 1F |  |  |
| 249686 | 13-Dec-16 |  | female |  | 2F 2M |  |
| 249687 | 13-Dec-16 |  | male | 1F |  |  |
| 249688 | 13-Dec-16 |  | male |  | 2M |  |
| 249691 | 13-Dec-16 |  | male |  | 1F 1M |  |
| 249692 | 13-Dec-16 |  | male |  | 2M |  |
| 256028 | 8-Nov-17 |  | male | 1F 1M | 1F | Y |
| 256030 | 8-Nov-17 |  | female | 1M |  | Y |
| 256031 | 8-Nov-17 |  | female | 1F | 1M | Y |
| 256032 | 8-Nov-17 |  | female | 1M |  | Y |
| 256038 | 8-Nov-17 |  | female | 1F | 1M | Y |
| 256042 | 8-Nov-17 |  | female |  | 1F | Y |
| 256045 | 8-Nov-17 |  | female | 2M | 1M | Y |
| 256047 | 8-Nov-17 |  | male | 1M | 1M | Y |
| 256048 | 8-Nov-17 |  | female | 1M |  | Y |
| 256049 | 8-Nov-17 |  | female | 1F |  | Y |
| 256050 | 8-Nov-17 |  | male | 1M |  | Y |
| 256051 | 8-Nov-17 |  | female | 1M |  | Y |
| 256052 | 8-Nov-17 |  | female |  |  | Y |
| 256053 | 8-Nov-17 |  | male | 1F |  | Y |
| 256054 | 8-Nov-17 |  | female | 1F | 1M | Y |
| 256055 | 8-Nov-17 |  | female | 1F | 1M | Y |
| 256061 | 8-Nov-17 |  | female |  | 3F | Y |
| 256062 | 8-Nov-17 |  | female |  | 1F 1M | Y |
| 256066 | 8-Nov-17 |  | female |  | 1M | Y |
| 249594 | 24-Nov-16 | Isla Panchito, near Chamela, Jalisco, MX | female |  | 1M 1F |  |
| 249595 | 24-Nov-16 |  | female | 2M |  |  |
| 249600 | 24-Nov-16 |  | female | 1M | 2M |  |
| 249601 | 24-Nov-16 |  | female | 2F | 1F |  |
| 249614 | 25-Nov-16 |  | female | 1M | 2M |  |
| 249630 | 28-Nov-16 |  | female | 1F 1M | 1F 2M |  |
| 249636 | 28-Nov-16 |  | female | 1F |  |  |
| 249642 | 29-Nov-16 |  | female | 1F |  |  |
| 255981 | 30-Oct-17 |  | male | 1M | 1M | Y |
| 255983 | 30-Oct-17 |  | female | 2F 1M | 1F 1M | Y |
| 255984 | 30-Oct-17 |  | female |  |  | Y |
| 255991 | 30-Oct-17 |  | male | 1M | 1M | Y |
| 255995 | 30-Oct-17 |  | female | 2M | 1M | Y |
| 255999 | 30-Oct-17 |  | female | 1F |  | Y |
| 256000 | 30-Oct-17 |  | female |  |  | Y |
| 255987 | 31-Oct-17 |  | female |  | 3F 1M | Y |
| 255988 | 31-Oct-17 |  | female | 2F | 1M | Y |
| 255997 | 31-Oct-17 |  | female | 1M | 1M | Y |
| 256002 | 31-Oct-17 |  | female | 1F | 2M | Y |
| 255986 | 5-Nov-17 |  | female |  | 1F | Y |
| 256012 | 5-Nov-17 |  | female |  |  | Y |
| 256013 | 5-Nov-17 |  | female |  |  | Y |
| 256014 | 5-Nov-17 |  | male |  |  | Y |
| 256015 | 5-Nov-17 |  | female |  |  | Y |
| 256016 | 5-Nov-17 |  | female |  |  | Y |
| 256029 | 5-Nov-17 |  | female | 1F 2M | 1F | Y |

Supplementary Figure 1 – Average relative abundance of samples clustered by sample type and sex, where applicable. In cave swabs and bat swabs, colors correspond to Class of bacteria, while in bat flies, colors correspond to genus of bacteria. *Bartonella* relative abundance is indicated with a bold black outline and *Wolbachia* relative abundance is indicated with a dashed black outline.


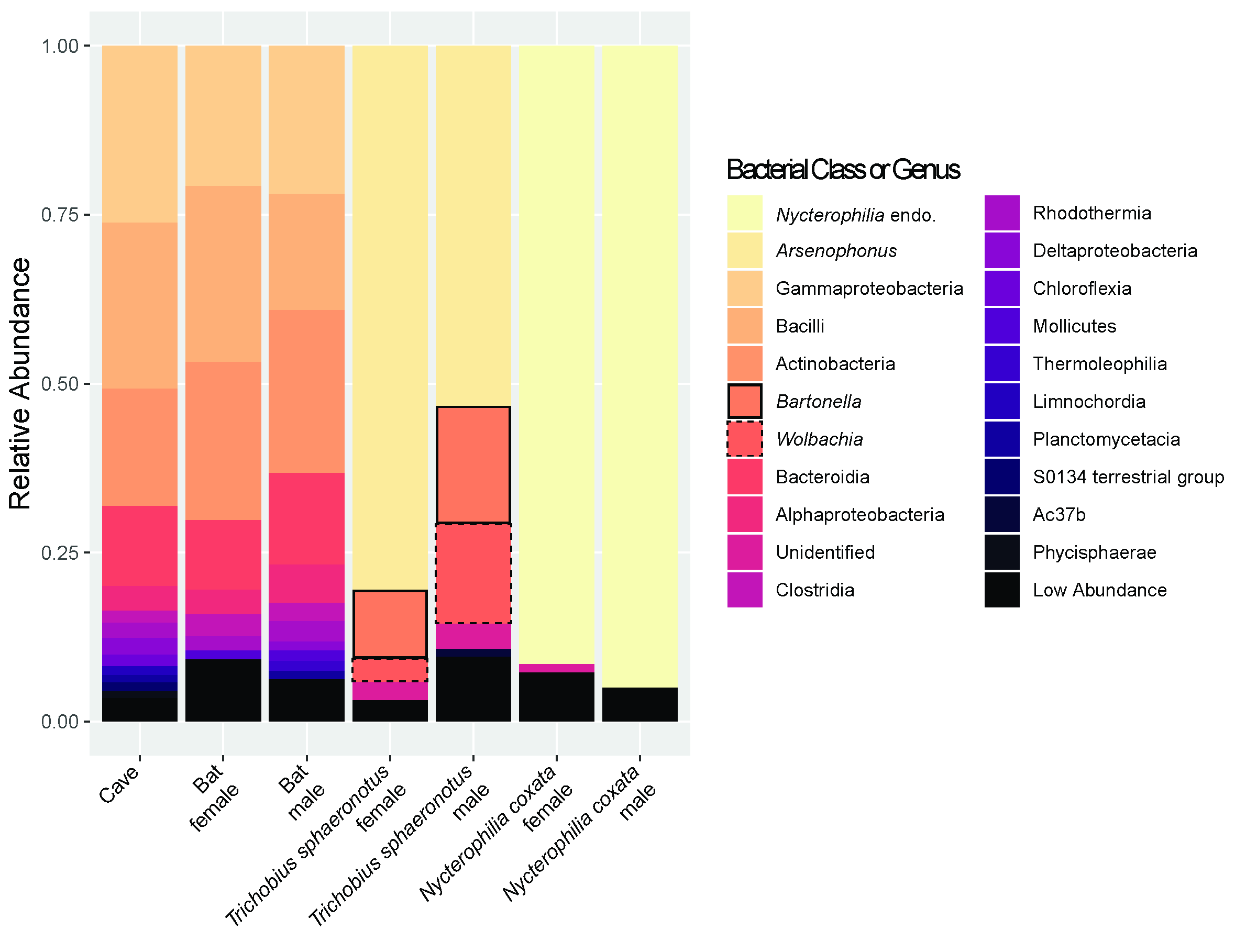


Supplementary Figure 2 – PCoA of *Nycterophilia coxata* samples only, with females indicated as circles and males indicated as triangles. Samples are colored by their *Bartonella* and *Wolbachia* infection status.


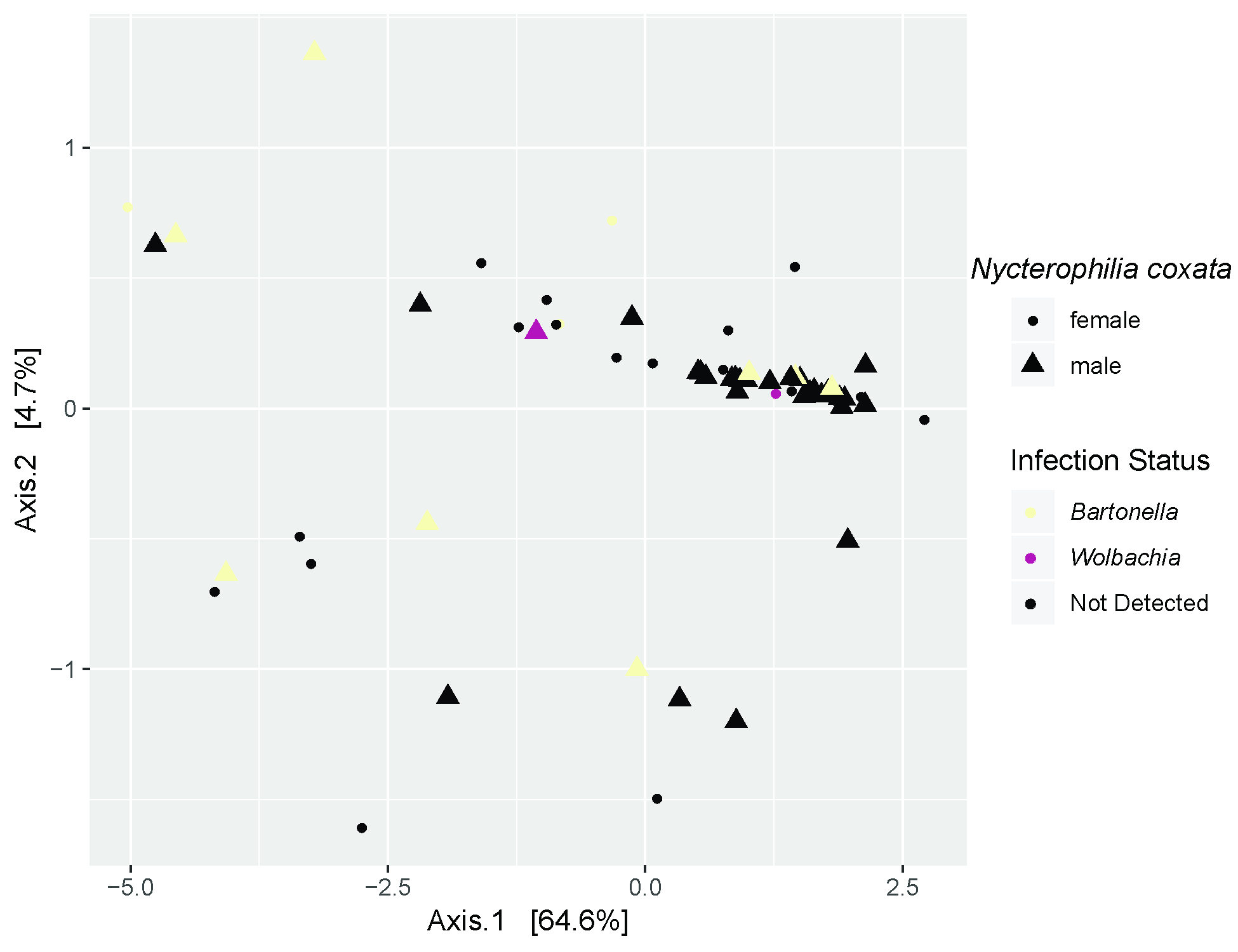


Supplementary Figure 3 – PCoA of *Trichobius sphaeronotus* samples only, with females indicated as circles and males indicated as triangles. Samples are colored by their *Bartonella* and *Wolbachia* infection status.


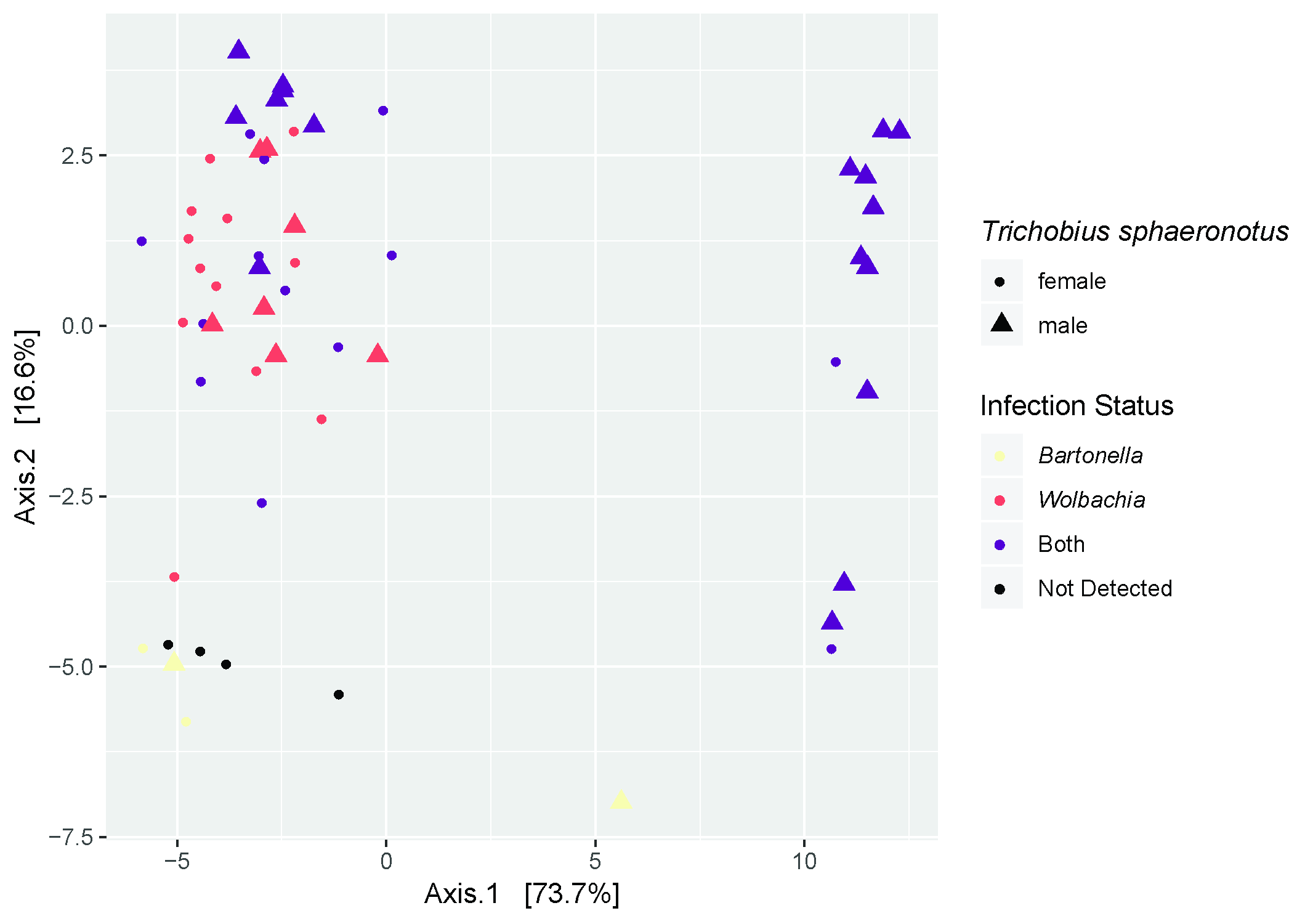


Supplementary Figure 4 – PCoA of microbiome samples colored by sample type, where all ASVs identified to *Arsenophonus* or *N. coxata*’s specific endosymbiont have been removed *in silico*. Ellipses indicate the 95% confidence interval for identifying a sample to a group, assuming a t distribution.


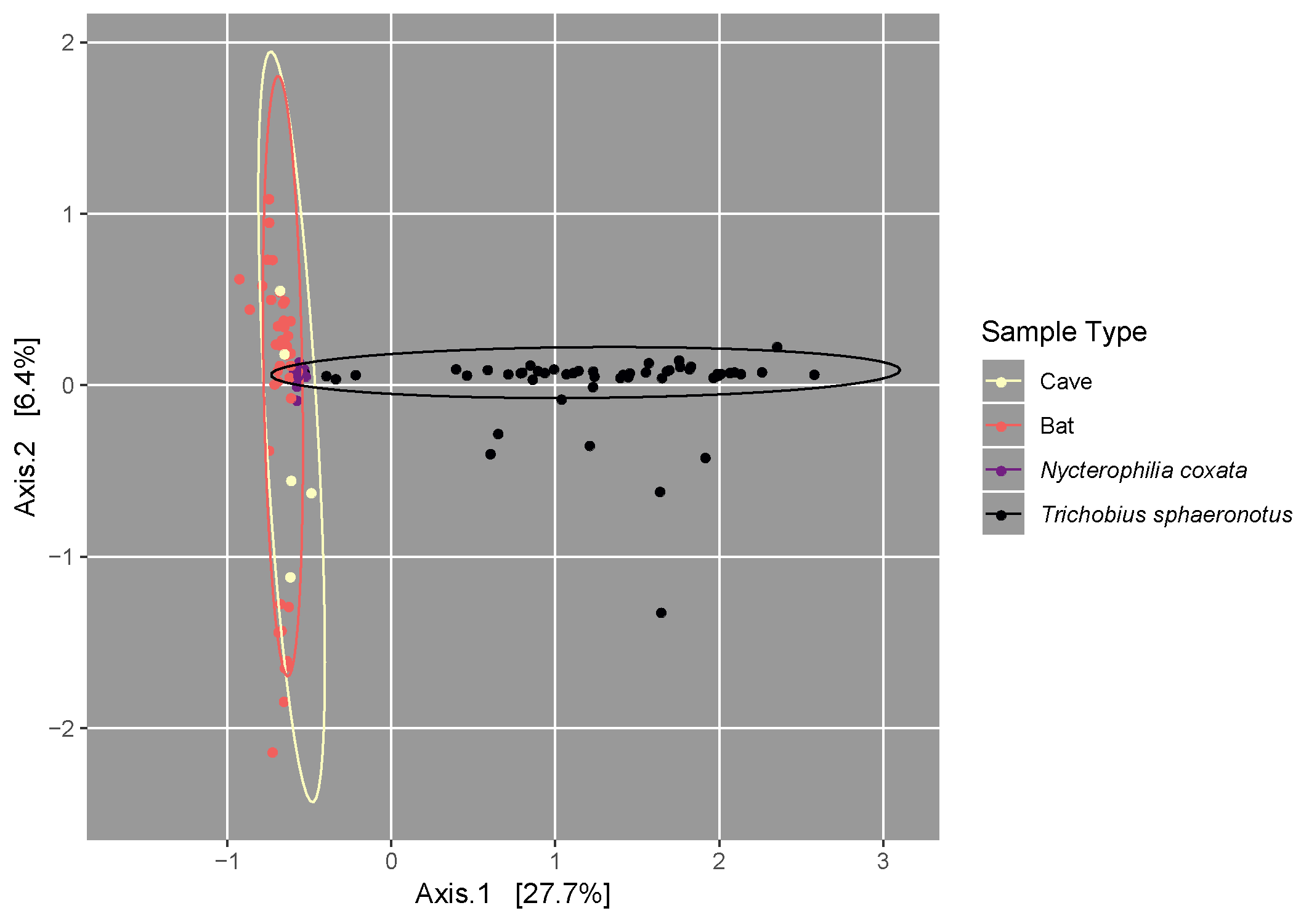

Supplement: Supplementary file 1 — Data S1 [file ECE3-15-e71120-s001.docx]
